# Annals of the Universal Sciences

**Published:** 1892-04

**Authors:** 


ANNUAL OF THE UNIVERSAL MEDICAL SCIENCES.—Edited by Charles E. Sajous, M. D., and Twenty Associate Editors. Five Volumes. Illustrated. Phila: F. A. Davis. 1891.

The fourth year of the "Annual" finds the work but little changed from the preceding year so far as the order and arrangement of its contents are concerned, which goes to show that the work has approached closely to perfection in these respects. It would indeed be difficult to suggest any improvement.

The work has maintained the high standard originally set for it, and the labor expended upon it has been done for the most part by the same able corps of editors who have made the previous issues so successful. The frequency with which the "Annual" is referred to in current medical literature is the best possible commentary upon its appreciation and success. Each department is compiled with great care, and increase of uniformity and improvement can be observed on every side, due to the increased practice and unceasing pains of the editors. As a whole, it is well designed, well executed, well illustrated, and so arranged as to be readily available to every person who desires to know of the work done in any field of medicine during the past year.

